# Omics Analysis of Blood-Responsive Regulon in *Bordetella pertussis* Identifies a Novel Essential T3SS Substrate

**DOI:** 10.3390/ijms22020736

**Published:** 2021-01-13

**Authors:** Jakub Drzmisek, Daniel Stipl, Denisa Petrackova, Branislav Vecerek, Ana Dienstbier

**Affiliations:** Laboratory of Post-Transcriptional Control of Gene Expression, Institute of Microbiology of the Czech Academy of Sciences, 14220 Prague, Czech Republic; drzmisek@biomed.cas.cz (J.D.); daniel.stipl@biomed.cas.cz (D.S.); petrack@biomed.cas.cz (D.P.)

**Keywords:** *Bordetella pertussis*, blood exposure, omics analyses, T3SS, gene expression, protein secretion

## Abstract

Bacterial pathogens sense specific cues associated with different host niches and integrate these signals to appropriately adjust the global gene expression. *Bordetella pertussis* is a Gram-negative, strictly human pathogen of the respiratory tract and the etiological agent of whooping cough (pertussis). Though *B. pertussis* does not cause invasive infections, previous results indicated that this reemerging pathogen responds to blood exposure. Here, omics RNA-seq and LC–MS/MS techniques were applied to determine the blood-responsive regulon of *B. pertussis*. These analyses revealed that direct contact with blood rewired global gene expression profiles in *B. pertussis* as the expression of almost 20% of all genes was significantly modulated. However, upon loss of contact with blood, the majority of blood-specific effects vanished, with the exception of several genes encoding the T3SS-secreted substrates. For the first time, the T3SS regulator BtrA was identified in culture supernatants of *B. pertussis*. Furthermore, proteomic analysis identified BP2259 protein as a novel secreted T3SS substrate, which is required for T3SS functionality. Collectively, presented data indicate that contact with blood represents an important cue for *B. pertussis* cells.

## 1. Introduction

Bacteria employ multiple systems to sense environmental stimuli and to adapt to the environmental niches they occupy. For bacterial pathogens, it is particularly important to respond appropriately to challenges associated with various environments within their hosts [1]. Therefore, pathogens sense specific cues associated with different host niches (e.g., temperature, pH, oxygen, iron, osmolarity) and integrate these signals to appropriately adjust the global gene expression [2,3,4,5,6].

*Bordetella pertussis* is a Gram-negative human pathogen that causes a highly contagious respiratory disease, whooping cough, aka pertussis. Upon infection, *B. pertussis* colonizes the ciliated epithelium of the upper respiratory tract in humans and causes inflammation, activation of immune responses and damage to host tissues [7]. To efficiently colonize the respiratory tract and evade the immune response, *B. pertussis* produces a wide variety of virulence factors [7,8,9]. Adhesins such as filamentous hemagglutinin, fimbriae and pertactin mediate attachment to ciliated respiratory epithelial cells and macrophages [7,9]. Furthermore, toxins including adenylate cyclase toxin, pertussis toxin and dermonecrotic toxin suppress and modulate host immune and inflammatory responses [10,11,12,13]. *B. pertussis* also encodes type III secretion system (T3SS), which is a known and important virulence factor in many other pathogenic bacteria, but studies on its role in *B. pertussis* pathogenesis are relatively scarce. Partially, this was because until recently, it was thought that in Tohama I, the laboratory-adapted reference strain of *B. pertussis*, T3SS is not functional. Nevertheless, later it was shown that in fresh clinical isolates as well as in Tohama I cells grown in iron- or glutamate-limited media or passaged in mice or human macrophages, the T3SS is operative [14,15,16,17,18]. Moreover, our recent studies employing highly sensitive LC–MS/MS technology proved that laboratory-adapted strain Tohama I secretes T3SS components also under standard laboratory conditions, though to a much lesser extent compared to fresh clinical isolates [19,20].

T3SS apparatus comprises a macromolecular injectisome that delivers effector proteins directly from bacterial cytosol into the host cell cytosol [21]. Thus far, only two effector proteins have been described in bordetellae. The BteA effector, also named BopC, is responsible for the cytotoxicity linked to T3SS, but its mechanism of action remains unknown [22,23,24]. Interestingly, an amino acid insertion within *B. pertussis* BteA protein renders this effector less cytotoxic compared to one produced by *B. bronchiseptica* [25]. The other effector is BopN protein, which, in spite of its homology with gatekeeper proteins [26], was reported to be translocated into the host cell nucleus and to modulate host immune response [27,28]. The T3SS genes are organized into two adjacent loci: *bsc* encoding the T3SS apparatus and *btr* encoding regulatory proteins [29,30,31], while the *bteA* gene, encoding the BteA effector, is expressed from a distant locus. The expression of T3SS genes in *B. pertussis* is controlled by the two-component system BvgAS, which regulates hundreds of genes, including those involved in virulence [32]. The membrane-bound sensor kinase BvgS phosphorylates the response regulator BvgA [32,33], which, in its phosphorylated form, activates the promoter of the extracytoplasmic function sigma factor BtrS encoded in *btr* locus. In turn, BtrS activates the expression of genes within the *btr* and *bsc* loci, though the expression of some genes within the *bsc* locus is not dependent on BtrS [30,32]. The activity of BtrS is inhibited by its cognate secreted antagonist BtrA and together, they comprise a regulatory mechanism coupling the expression and function of T3SS [31]. In addition, T3SS is regulated post-transcriptionally by three proteins orthologous to partner switching proteins: BtrV is required for T3SS protein translation and/or stability, whereas BtrU and BtrW are necessary for secretion [30]. Another layer of regulation is added by the RNA chaperone Hfq, an important post-transcriptional regulator of Gram-negative bacteria, which was shown to be required for the expression and functionality of T3SS in *B. pertussis* [16,19]. As mentioned above, the expression of the T3SS apparatus can be induced upon contact with the host [15,16,34,35] or by nutrient limitation [17,18]. Furthermore, it was reported that the activity of T3SS is increased in the presence of CO_2_ [36] or upon exposure to blood or serum [37]. On the other side, the intramacrophage expression of T3SS genes in *B. pertussis* and *B. bronchiseptica* cells is reduced [38,39].

In this work, we employed omics techniques to characterize the global transcriptional response of *B. pertussis* cells exposed to blood, as well as changes in gene expression and protein-secretion profiles upon loss of contact with blood. Importantly, our data revealed that direct exposure to blood has a dramatic effect on global gene expression profiles in *B. pertussis* and that contact with blood is a prerequisite for efficient secretion of T3SS components.

## 2. Results

### 2.1. Contact with Blood Augments the Expression and Secretion of T3SS Components in B. pertussis

**Figure 1:**
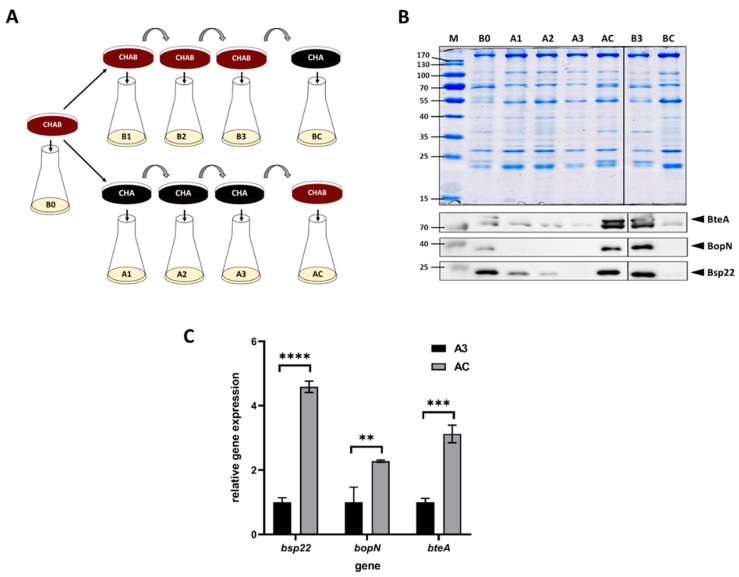
Blood exposure induces the expression and secretion of T3SS substrates in *B. pertussis*. (**A**) Scheme depicting the experimental layout and origin of samples used for Western blot and RT–qPCR analyses of cells grown in liquid cultures inoculated either from charcoal plates supplemented with blood (CHAB) or plain charcoal plates (CHA) plates. Cells from liquid cultures were used for RT–qPCR (pellets) and Western blot (supernatants) analyses. (**B**) Samples of precipitated proteins equivalent to 1 OD_600_ unit were separated on 12.5% SDS–PAGE gel (upper panel) and analyzed by immunoblotting using antibodies against BteA, BopN and Bsp22 proteins. Only relevant parts of the membranes are shown; samples B1 and B2 are not shown. The result is representative of three experiments. Positions of BteA, BopN and Bsp22 proteins on the membranes are depicted with arrowheads. (**C**) RT–qPCR analysis of relative expression of *bsp22*, *bopN* and *bteA* genes. Relative gene expression was compared between cells harvested from culture AC (inoculated from a plate containing blood) and culture A3 (inoculated from a plate lacking blood), which served as the control (relative expression was set to 1). Differences between samples were statistically tested by multiple comparison test using the Bonferroni’s method; **, adj. *p*-value < 0.01, ***, adj. *p*-value < 0.0004, ****, adjusted *p*-value < 0.0001.

A previous study revealed that the expression of T3SS in *B. bronchiseptica* and *B. pertussis* is upregulated in response to blood added to cells pre-grown in liquid medium [37]. Nevertheless, since the liquid cultures of *B. pertussis* are inoculated from agar plates containing blood, we asked whether the presence of blood in a solid medium would modulate the T3SS expression as well. To answer this question, we used charcoal agar, which supports the growth of *B. pertussis* also in the absence of blood. Thus, we passaged *B. pertussis* on plain charcoal plates (CHA) or on charcoal plates supplemented with blood (CHAB), and then we used these plates to inoculate liquid cultures to monitor the expression and secretion of selected T3SS components. As schematically depicted in Figure 1A, *B. pertussis* cells were initially grown on a CHAB plate containing blood and used to inoculate the liquid culture B0. Next, cells from the master CHAB plate were streaked on the new CHAB plate (culture B1) as well as on the CHA plate (culture A1). *B. pertussis* cells were then passaged twice onto plates of the same type (cultures B2 and B3 or A2 and A3, respectively). Finally, cells from the CHAB plate were passaged onto the CHA plate (culture BC) and vice versa (culture AC). From each liquid culture, samples were taken to determine the expression of the T3SS genes *bopN*, *bsp22* and *bteA* and secretion of their corresponding products. Western blot analysis (Figure 1B) showed that when the cultures were inoculated from plates supplemented with blood, the levels of secreted BopN, Bsp22 and BteA proteins remained unchanged. In contrast, cells passaged on plates lacking blood gradually lost the ability to secrete tested T3SS proteins. As also shown in Figure 1B, passaging of cells from CHA plate onto CHAB plate (culture AC) resulted in reactivation of secretion of T3SS proteins, while the transfer of cells from CHAB plate onto CHA plate yielded highly reduced secretion of T3SS components (culture BC). To check whether these effects resulted from an altered expression of the corresponding genes and to validate the inducing effect of blood, we compared the expression of *bopN*, *bsp22* and *bteA* genes in cells harvested from cultures AC and A3. When compared to A3 culture, the expression of the tested genes in cells harvested from culture AC increased roughly 2- to 5-fold (Figure 1C).

**Figure 2:**
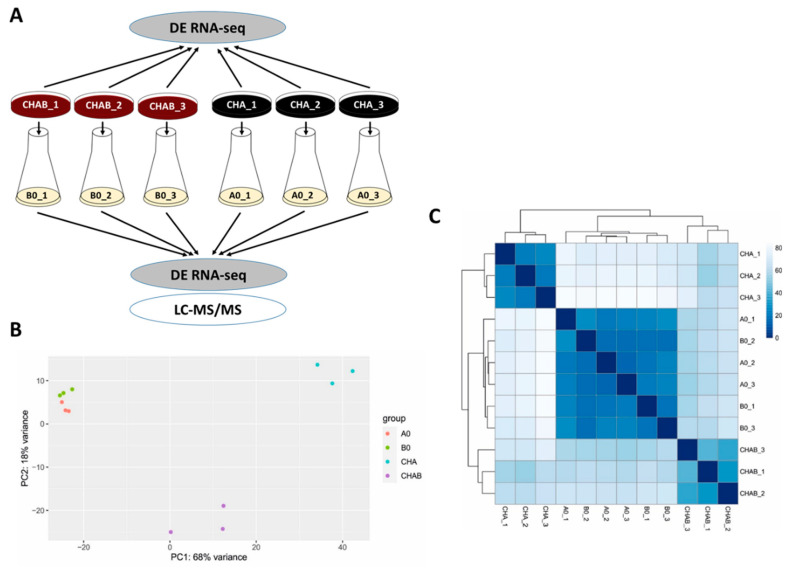
Direct exposure to blood rewires the global expression profiles in *B. pertussis*. (**A**) Scheme depicting the experimental layout and origin of samples used for transcriptomic and proteomic profiling of cells grown in the presence (CHAB and B0 samples) or absence (CHA and A0 samples) of blood. Cells were either harvested directly from CHAB and CHA plates for RNA-seq analysis or were used to inoculate B0 and A0 liquid cultures, respectively. Cells from liquid cultures were used for RNA-seq (pellets) and LC–MS/MS (supernatants) analyses. (**B**) Principal component analysis of samples based on RNA-seq analysis. (**C**) Heatmap showing hierarchical clustering of RNA-seq data by Euclidian sample-to-sample distances (represented by the color gradient).

This experiment indicated that blood present in the agar plate induces expression and secretion of T3SS components and encouraged us to test the global effects of blood exposure on gene expression profiles in *B. pertussis*. Towards this aim, we chose two different strategies (see Figure 2A). First, to determine the direct effects of blood on gene expression, we analyzed biological triplicates of cells harvested directly from charcoal agar plates supplemented with blood (CHAB_1 to CHAB_3) and from plates lacking blood (CHA_1 to CHA_3). Second, we used the cells from the same plates to inoculate liquid cultures (B0_1 to B0_3 and A0_1 to A0_3, respectively). The analysis of cells grown in SS medium allowed us to find out whether the blood-modulatory effects are waning in the absence of blood as observed with T3SS and, importantly, how previous incubation in the presence of blood affects the protein secretion. Total RNA was purified from cells harvested directly from plates (CHAB_1-3 and CHA_1-3) or from liquid cultures (B0_1-3 and A0_1-3) and used for RNA-seq analysis. Furthermore, proteins precipitated from filtered culture supernatants were identified and quantified by LC–MS/MS method. The RNA-seq analysis yielded, on average, approximately 47 million read pairs per sample, which mapped to *B. pertussis* transcriptome. Principal component analysis (PCA) of RNA-seq data revealed that all samples could be roughly separated into three clusters: (i) cells grown on plates supplemented with blood, (ii) cells grown on plates without blood and (iii) cells subcultured in liquid medium regardless of the presence of blood in the inoculation plate (Figure 2B). Hierarchical clustering of all samples was in line with the PCA data as it indicated that the presence of blood leads to the clear separation of cells harvested directly from plates while cells collected from liquid cultures cluster together and, apparently, display less prominent differences (Figure 2C).

### 2.2. Direct Exposure to Blood Substantially Rewires the Global Gene Expression in B. pertussis

**Figure 3:**
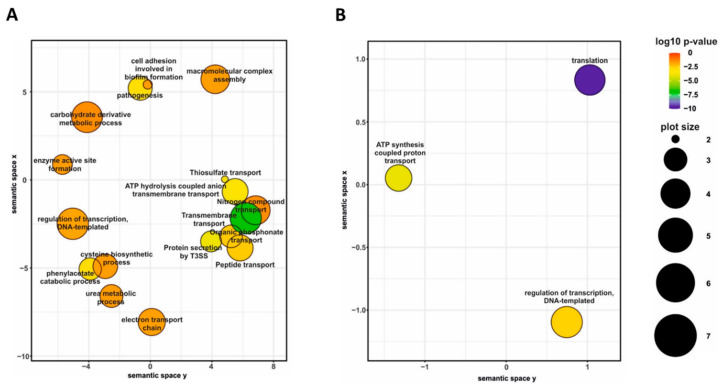
Gene ontology (GO) term enrichment analysis of *B. pertussis* genes modulated by blood. Significantly enriched GO terms from the domain “biological processes” were identified within gene sets either upregulated (**A**) or downregulated (**B**) in response to direct blood exposure. Results were summarized and visualized by REVIGO as a scatter plot. Circle size depicts the number of genes associated with the respective category; colors encode the significance level of the enrichment.

Differential gene expression analysis of cells harvested from agar plates revealed that the presence of blood in charcoal agar plates resulted in significant modulation (|log_2_FC| > 1; q-value < 0.05) of 690 genes, including 36 non-coding RNA and one transfer RNA genes (Table S1). Among the differentially expressed (DE) genes, 373 genes were downregulated, and 317 were upregulated in the presence of blood. Given the large number of DE genes, we used gene ontology (GO) term enrichment analysis for all DE genes to get overall insight into the functional profiles of blood-responsive genes. A large portion of the GO terms significantly enriched among the DE genes, which were upregulated in response to blood, was associated with cellular transport and metabolism such as “transmembrane transport”, “peptide transport”, “nitrogen compound transport”, “cysteine biosynthetic process” and “carbohydrate derivative metabolic process”. Indeed, several operons coding for sugar and amino acid ATP-binding cassette transporters (*BP0119-BP0122*, *BP1273-BP1277*, and *BP3568-BP3577*) as well as for tripartite tricarboxylate transporters (*BP0649*, *BP1675*) were among the most highly upregulated genes. Importantly, biological processes “pathogenesis” and “protein secretion by T3SS” were also enriched among the upregulated genes (Figure 3A) primarily due to increased expression of 12 genes belonging to *bsc* locus encoding the T3SS apparatus (*BP2246*-*BP2254* and *BP2258*-*BB2260*).

On the other side, the analysis revealed that blood exposure causes downregulation of the genes associated with global cellular processes, such as “ATP synthesis coupled proton transport” and “translation” (Figure 3B). For example, many genes encoding ribosomal proteins (including almost all genes within the *rpsL*-*rpoA* cluster) and elongation factors G and Tu were downregulated in response to contact with blood. Finally, the GO term “regulation of transcription, DNA-templated” was enriched in both up- and downregulated genes (Figure 3). Indeed, the expression of almost 50 transcriptional regulator genes was significantly modulated in response to contact with blood. These observations highlighted the immense extent of changes in *B. pertussis* global regulatory networks associated with direct exposure to blood.

In addition, our analysis identified several gene categories that were not captured by the GO term enrichment analysis, such as those linked to bacterial attachment and to iron metabolism (Table S1). Gene coding for serotype 3 fimbrial subunit (*fim3*) was 10-fold down-regulated in the presence of blood and thereby belonged to one of the most highly impacted genes. Likewise, genes coding for fimbrial adhesin FimD and filamentous hemagglutinin transporter protein FhaC were significantly downregulated in the presence of blood. Furthermore, several genes involved in iron transport and metabolism were significantly downregulated, e.g., those encoding ferric siderophore receptors BfrB, BfrF and BfrI, TonB-dependent iron receptor BP0857, iron-binding proteins IRP1-3 and BP1139, bacterioferritin BP0546, heme uptake protein HurI as well as various iron–sulfur cluster proteins including a putative regulatory protein BP1155 containing 4Fe-4S cluster.

### 2.3. Most of the Blood-Mediated Effects Are Lost in Liquid Media Lacking Blood

Next, we tested whether the changes in global gene expression resulting from direct exposure to blood endure upon cultivation in growth media lacking blood. Thus, we performed differential gene expression analysis of *B. pertussis* cells, which were inoculated from charcoal agar plates with (CHAB) and without (CHA) blood and subsequently cultured in SS medium (B0 and A0 cultures, respectively). Compared to the effect seen in cells harvested directly from plates, the number of significantly deregulated genes (|log_2_FC| > 1; q-value < 0.05) was dramatically reduced. Indeed, previous exposure to blood modulated the expression of only 18 genes in cells harvested from liquid cultures (Table 1).

Nevertheless, these results were in good agreement with our PCA data as they indicated that cells subcultured in SS medium display highly similar gene expression profiles regardless of the contact with blood. A large portion of upregulated genes (6 out of 16) was related to the T3SS injectisome, including *bteA*, *bopB*, *bopD* and *bsp22* genes encoding its secreted components. Among other upregulated genes, we found *putA* encoding trifunctional proline dehydrogenase, alanine racemase gene *alr* lying within the *btr* locus and, interestingly, also a putative sRNA (Candidate_Transcript_071), which was predicted to be located immediately downstream of the *bteA* gene [40]. These results suggested that in contrast to the vast majority of blood-responsive genes, several T3SS genes remained to be modulated in liquid medium lacking blood. Next, we tested how this phenomenon translated into protein-secretion profiles.

**Figure 4:**
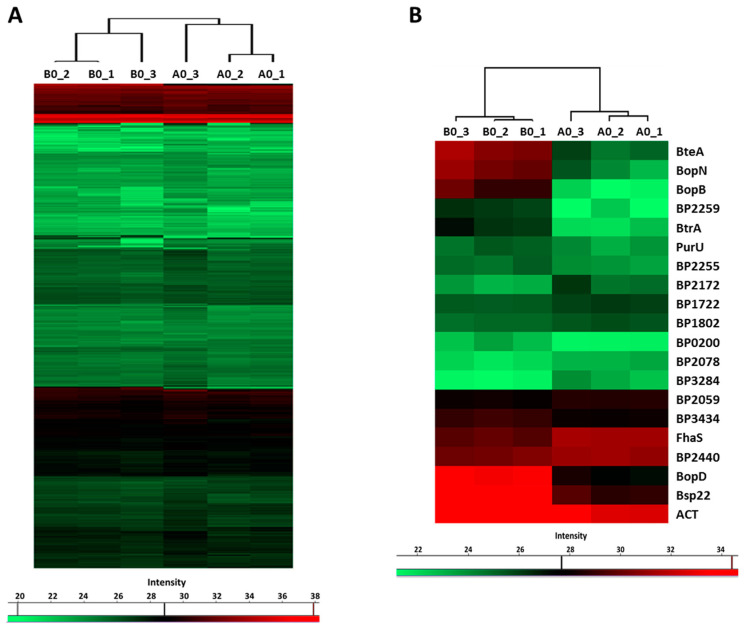
Previous exposure to blood has only a limited impact on protein secretion. Proteins were isolated from supernatants of cultures inoculated from CHAB plates (B0 cultures) and from CHA plates (A0 cultures). (**A**) Heatmap showing hierarchical clustering performed on label-free quantification (LFQ) intensity values of all 751 secreted proteins which passed the detection criteria. (**B**) Heatmap showing hierarchical clustering of all significantly (q-value < 0.1) differentially secreted proteins. Color-coded scale bars denote the LFQ intensities of identified proteins.

LC–MS/MS analysis of the proteins precipitated from the culture supernatants identified 751 proteins, whose label-free quantification (LFQ) intensities passed our detection criteria. Hierarchical clustering analysis revealed that global profiles of secreted proteins, identified in cells inoculated from plates with (B0 samples) and without the blood (A0 samples), are highly similar. Nevertheless, B0 and A0 samples still clustered apart and thereby indicated that some proteins were differentially secreted (Figure 4A). Indeed, based on the *t*-test, 20 proteins were significantly differentially secreted in response to blood present in agar plates (Figure 4B). Notably, the abundance of several T3SS substrates such as BopD, BopN, BopB, Bsp22 and BteA was strongly enhanced (Table 2). Importantly, among blood-responsive proteins, shotgun proteomics also identified the BtrA protein, a secreted anti-sigma factor, which has not been shown to be secreted by *B. pertussis* cells so far. Furthermore, secretome analysis revealed a novel T3SS substrate, BP2259 protein, which is an uncharacterized protein encoded within the *bsc* locus. The abundance of adenylate cyclase toxin was also increased, and it suggested that secretion of this important virulence factor is also induced by blood. Collectively, these results confirmed our RNA-seq data and indicated that with the exception of a few genes, the effect of blood is rather transient, and once the contact with the blood is lost, bacterial cells promptly adjust their gene expression to new conditions.

### 2.4. Transcriptional Dynamics of the T3SS Genes

**Figure 5:**
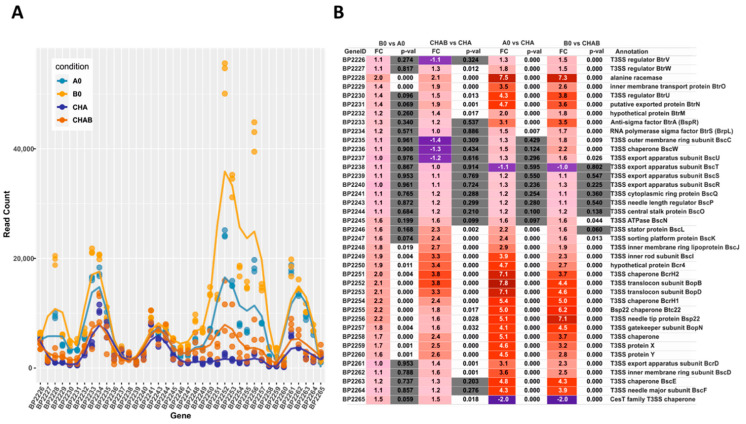

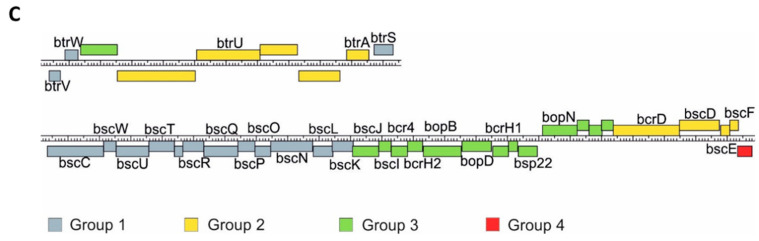
Transcriptional response to blood exposure is not uniform among T3SS genes. The expression of genes within *bsc/btr* loci was analyzed in cells that were either harvested directly from CHAB and CHA plates or from B0 and A0 liquid cultures. (**A**) Graph representing normalized read count for each gene within *bsc/btr* loci. Dots show the read count in triplicates of samples grown under four different conditions. Lines represent the smoothed averages of the read counts in triplicates. (**B**) Differential gene expression analysis of *bsc/btr* loci. Gradient represents fold changes of gene expression between different conditions. Blue and red colors represent downregulation and upregulation of corresponding genes; respectively, color intensity indicates the extent of the modulation. Cells filled with gray color denote that the change in expression of the corresponding gene is not significant (*p*-value > 0.05). (**C**) Grouping of T3SS genes within *btr* (upper panel) and *bsc* (lower panel) loci according to their gene expression profiles under different growth conditions. Genes are depicted as color-coded blocks based on the affiliation to the corresponding group; their position indicates the strandedness.

Our transcriptomic and proteomic analyses revealed that the expression and secretion of T3SS components were significantly increased in the cells exposed to blood. These results also indicated that not all the genes within *bsc*/*btr* loci respond to blood to the same extent. Therefore, we examined the expression of T3SS genes in more detail. For that, we analyzed the normalized read counts (Figure 5A) and changes in gene expression (Figure 5B) for all T3SS genes in cells treated or untreated with blood and harvested from solid or liquid media. The outcome of these analyses revealed that based on their transcriptional profiles, the T3SS genes fell roughly into four groups (Figure 5C). In the first group, comprising genes *BP2226*, *BP2227* and *BP2234*-*BP2247* (e.g., *btrS* and *bscN*), the read counts remained relatively constant and thereby indicated that neither the type of the medium nor the presence of blood affected their expression. The second group comprised genes *BP2229* to *BP2233* (e.g., *btrA* and *btrU*) and *BP2261* to *BP2264* (e.g., *bscE*, *bscF*), whose expression was affected predominantly by the type of the growth medium, irrespective of the exposure to blood. The expression of genes within the third group, such as *BP2228* and *BP2248* to *BP2260* (e.g., *bopB*, *bopD*, *bopN* and *bsp22*), was modulated by both the type of the growth medium and exposure to blood. The last group contained only one gene, *BP2265*, encoding a hypothetical T3SS chaperone. In contrast to all other T3SS genes, its expression was significantly downregulated upon passaging of cells from plates to liquid media (Figure 5B). Intriguingly, these results indicated that compared to solid agar plates, the expression of the majority of T3SS genes is increased in liquid medium, and only some T3SS genes are responsive to blood.

### 2.5. The Product of the BP2259 Gene Is Required for Secretion of T3SS Component Bsp22

**Figure 6:**
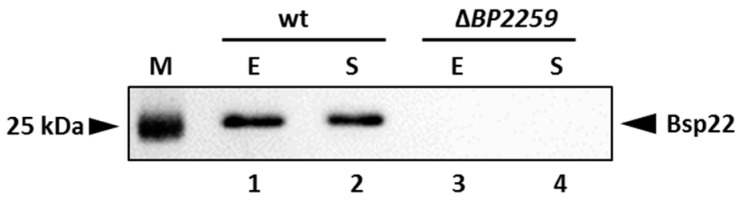
Product of the *BP2259* gene is required for secretion of T3SS protein Bsp22. Proteins were precipitated from culture supernatants of Tohama I strain (lanes 1 and 2) and its isogenic ∆*BP2259* mutant (lanes 3 and 4) grown to exponential (lanes 1 and 3; E) or stationary (lanes 2 and 4; S) phases of growth. Samples equivalent to 1.0 OD_600_ units were separated on 12.5% SDS–PAGE gel and analyzed by immunoblotting using antibodies against Bsp22 protein. The only relevant part of the membranes is shown. The result is representative of three experiments. Positions of the Bsp22 protein and 25 kDa protein of the size marker (M) on the membrane are depicted with arrowheads.

Our proteomic data identified a novel secreted T3SS substrate, a product of the *BP2259* gene. Homology search revealed that this protein shares 27.1% and 30.4% homology with the products of T3SS *yscX* and *pcr3* genes of *Yersinia enterocolytica* and *Pseudomonas aeruginosa*, respectively [41,42]. The exact role of these genes in T3SS functionality is not clear. However, in both pathogens, the deletion of the *BP2259* homolog resulted in defective secretion of T3SS substrates. Therefore, we decided to construct a markerless Δ*BP2259* deletion mutant in *B. pertussis* and test its ability to secrete T3SS components. As shown in Figure 6, the strain lacking the *BP2259* gene did not secrete Bsp22 protein into the medium and thereby manifested that the product of the *BP2259* gene is required for T3SS functionality.

## 3. Discussion

The ability of a pathogen to sense and adapt to the host environment is instrumental for its survival. Therefore, identification of the genes responsive to various host signals and environmental cues is important for our understanding of infection processes. Blood and its components likely belong to the cues that *B. pertussis* encounters in the host, and it may serve as a specific sign of the host tissue inflammation and damage. Indeed, recent DNA microarray analysis published by Gestal et al. [37] revealed that the addition of blood or serum triggers substantial alterations in gene expression profiles of *B. bronchiseptica*, a close relative of *B. pertussis*. Moreover, the study showed that in *B. pertussis*, serum exposure induces the expression of several T3SS genes and adenylate cyclase gene *cyaA* and increases cytotoxicity against macrophages. Nevertheless, the global response to blood was not addressed. Therefore, we applied RNA-seq and LC–MS/MS methods to study the global transcriptional and proteomic responses to blood exposure in *B. pertussis*. First, we studied the immediate effects of blood in cells grown on solid charcoal agar. Second, we tested the possibility that cells inoculated from agar plates containing or lacking blood display different transcriptomic and proteomic profiles when cultivated in liquid medium lacking blood. Furthermore, the absence of blood, containing large amounts of proteins such as albumin, allowed us to analyze the amounts of secreted proteins in culture supernatants.

Here presented study unveiled that direct exposure to blood triggers a huge response in *B. pertussis* as the expression of almost 20% of the genome was significantly modulated. While the blood responsive stimulon in *B. bronchiseptica* comprised 89 genes [37], our RNA-seq analysis identified 690 significantly differentially expressed genes in *B. pertussis*. While it is difficult to compare two pathogens occupying different niches using two different transcriptomic approaches, both analyses identified T3SS genes as a part of blood-responsive regulon. Furthermore, omics analyses of cells exposed to blood and further subcultured in liquid media without blood clearly documented that upon loss of contact with blood, the expression levels of the vast majority of genes went back to the levels observed in unexposed cells. In line with this modest change in gene expression profiles, only 20 proteins, including seven T3SS substrates, displayed significantly different levels in culture supernatants in response to previous blood treatment.

Our results show that upon direct exposure to blood, *B. pertussis* undergoes adaptive metabolic shift as numerous genes linked to transport and metabolism of various compounds were significantly deregulated. Most prominent changes in expression were observed for genes associated with the transport and metabolism of sugars and amino acids. Similar results were also obtained with other pathogens exposed to blood, such as *Staphylococcus aureus*, *Streptococcus agalactiae*, group A *Streptococcus* and *Enterococcus faecalis* [3,5,43,44]. Therefore, the blood-triggered changes in the central metabolism can be seen as an adaptive response to highly abundant carbohydrates and amino acids present in the blood. Furthermore, our data suggest that the observed metabolic shift is accompanied by a slowdown of protein synthesis. Indeed, several genes coding for ribosomal proteins were downregulated in the presence of blood. In agreement with our results, ribosomal genes were also downregulated in response to blood in *B. bronchiseptica* [37].

Despite being rich in organic nutrients, blood is a very poor source of iron as the majority of blood iron is sequestered by various iron-binding proteins [45]. Bacterial pathogens developed different systems to scavenge the iron, and many of those systems were shown to be upregulated during incubation in blood [3,43,44,46,47]. The upregulation of iron acquisition genes in response to blood and serum exposure was also documented in *B. bronchiseptica* [37]. Surprisingly, our results showed that many ferric siderophore receptors, as well as iron-binding and iron uptake proteins, were significantly downregulated in *B. pertussis* cells grown on charcoal plates containing blood. We speculate that our observations may result from partial lysis of red blood cells present in agar, thereby providing the pathogen with sufficient levels of iron from released hemoglobin. The exposure to blood also affected the expression of several genes involved in virulence. Our results indicate that genes coding for fimbrial proteins are significantly downregulated during the growth of *B. pertussis* on blood agar plates. Due to their high immunogenicity, fimbriae are included in several pertussis vaccine formulations [48]. Of note, fimbriae have been reported to activate human peripheral blood monocytes [49]. Thus, it is possible that upon contact with damaged tissues within the host, the reduction of fimbriae production may assist pathogen in immune evasion and thereby augment its survival during infection.

In line with a previous report [37], blood-responsive regulon included the *bsc/btr* loci encoding the T3SS regulators, substrates and structural proteins. The link between blood exposure and increased expression of T3SS genes has also been reported in *Burkholderia cenocepacia* during its transition from lungs to the bloodstream in patients with cystic fibrosis [50]. Notably, our results unveiled several interesting aspects of blood-mediated modulation of T3SS expression in *B. pertussis*.

First, we showed that T3SS genes remained upregulated when the cells were subcultured in liquid media lacking blood. Apparently, induced expression of T3SS genes in *B. pertussis* cells exposed to blood can be “memorized” as these genes remained upregulated when this cue was removed from the environment. Such a transcriptional memory has already been described as an important adaptive mechanism linked to sugar metabolism [51], and it was suggested that this strategy might allow bacteria to elicit quick transcriptional response once the cue or metabolite is available again. To our knowledge, there is no other example of transcriptional memory linked to the expression of virulence factors. However, it should be noted that repetitive passaging of cells in the absence of blood resulted in reduced secretion of T3SS substrates.

Second, the response to blood exposure or to changes in the type of cultivation medium was not uniform within *bsc*/*btr* loci. The expression of some genes remained constant, other genes were modulated only in response to change from solid to the liquid medium, but a large portion of genes responded to both change of the medium and blood exposure. For example, the expression of regulatory *btrV*, *btrW* and *btrS* genes were comparable in all tested conditions, while the expression of two other regulators, *btrU* and *btrA,* was significantly increased in cells harvested from liquid cultures irrespective of their previous exposure to blood. Notably, the expression of genes coding for secreted components (e.g., *bopB*, *bopD*) was induced in liquid cultures and in response to contact with blood.

The third observation indicated that the inducing effect of blood exposure determined in broth-grown cells was further amplified at the secretion level as illustrated by dramatically increased levels of T3SS substrates, including BP2259 and BtrA proteins. The identification of BtrA protein by LC–MS/MS technique represents an important observation as the secretion of BtrA in *B. pertussis* cultures has not been reported as yet. Intriguingly, our data suggest that in *B. pertussis*, the BtrA plays a different role in the regulation of T3SS expression than that described in *B. bronchiseptica* strains S798 and RB50 [31,52]. In these strains, BtrA was identified as an antagonist of alternative sigma factor BtrS, which is required for transcription of the secreted components encoded within the *bsc* locus [31,52]. However, our study demonstrated that upon transfer of *B. pertussis* cells from solid to the liquid medium, the enhanced expression of the negative regulator *btrA* was paralleled by an increased expression of T3SS genes. This was surprising, especially for cells grown in cultures inoculated from bloodless plates as these cells expressed, but did not secrete BtrA efficiently and, as a consequence of high concentrations of cytosolic BtrA, the expression of BtrS-dependent genes should have been reduced. Thus, these results suggested that BtrAS regulatory circuit is more complex than previously thought. Interestingly, a comparison of BtrA proteins in all completely sequenced *B. pertussis* and *B. bronchiseptica* strains available in GenBank showed that some *B. bronchiseptica* strains (including RB50 and S798) display substantial differences in N-terminal parts of BtrA proteins compared to *B. pertussis* and several other *B. bronchiseptica* strains, majority of which, intriguingly, was of human origin (Figure S1). It is tempting to speculate that observed differences in sequences of BtrA proteins resulted from the adaptation of *B. pertussis* and some *B. bronchiseptica* strains to the human host to facilitate the optimal function of the BtrA protein.

In the course of this study, we identified BP2259 protein as a novel T3SS substrate, and furthermore, we showed that it is required for the secretion of T3SS protein Bsp22 in *B. pertussis*. Thus, it will be of high importance to further investigate how the blood-responsive protein BP2259 contributes to T3SS functionality in *B. pertussis*. The apparent discrepancy between the effects of blood exposure on gene expression and protein secretion suggested that T3SS expression is also controlled at the post-transcriptional level. Recently, we have observed that deletion of the *hfq* gene in *B. pertussis* resulted in highly reduced secretion of T3SS components relative to gene expression levels [16]. RNA chaperone Hfq is a key player in small non-coding RNA (sRNA)-mediated post-transcriptional control of gene expression [53] and an essential factor of virulence in Gram-negative pathogens [54,55]. We have shown that Hfq is required for virulence [56] and for T3SS functionality in *B. pertussis* [16]. Therefore, we were tempted to determine whether any of the sRNAs identified in *B. pertussis* Tohama I strain [40,57,58] would explain the positive effects of blood and Hfq on the secretion of T3SS components. In the current study, we identified only one sRNA, Candidate_transcript_071, to be significantly differentially expressed in response to previous blood exposure. Interestingly, our previous study revealed that the expression of this sRNA is downregulated in the *hfq* mutant, though not significantly (log_2_FC of −0.79) [19]. Considering that this sRNA is transcribed either next to or within 3’UTR of the *bteA* gene [40] and its expression is positively modulated by blood and Hfq, it will be of our primary focus to investigate the potential involvement of this sRNA in T3SS functionality in our future studies.

In summary, in this study, we demonstrated that in response to direct blood exposure, *B. pertussis* cells adapt their global gene expression profiles. Cells treated with blood activate several pathways involved in nutrient transport and metabolism as well as several transcription regulators and virulence genes, including the T3SS apparatus. While for invasive bacterial pathogens, such as *Staphylococcus* sp. [3], *Enterococcus faecalis* [44], *Streptococcus* sp. [5] and *Neisseria meningitidis* [46], entry into the bloodstream denotes an important stage of infection, *B. pertussis* does not typically cause invasive disease. Overall, there have been only a few reports describing *B. pertussis* bacteremia in immunocompromised people [59,60,61]. Nevertheless, our results indicate that contact with blood represents an important cue for *B. pertussis* cells, and our knowledge on how this pathogen responds to this cue is crucial for understanding *B. pertussis* pathogenesis in the host.

## 4. Materials and Methods

### 4.1. Growth Conditions and Sample Collection

The *Bordetella pertussis* Tohama I strain [62] was grown either on charcoal agar (CHA) or on charcoal agar supplemented with 15% defibrinated sheep blood (CHAB) for 3 to 4 days at 37 °C. For liquid cultures, bacteria were grown in Stainer–Scholte (SS) medium [63] supplemented with 0.1% cyclodextrin and 0.5% casamino acids at 37 °C. To harvest cells for RNA isolation directly from agar plates, cells were scraped from the plates, shortly washed in SS medium and pelleted by centrifugation (14,000× *g*, 4 °C, 10 min). For isolation of RNA and secreted proteins from broth-grown bacteria, cells were first inoculated from CHA and CHAB plates and grown overnight in SS medium to the late exponential phase of growth (OD_600_ ≈ 2.0). Next, cells were pelleted by centrifugation (14,000× *g*, 4 °C, 10 min) for RNA isolation and corresponding culture supernatants were filtered through 0.22 μm filters and precipitated for secretome analysis. Three independent cultivation experiments were performed to collect three biological replicates for RNA and protein isolation.

### 4.2. RNA Isolation

Cell pellets were suspended in TE buffer (10 mM Tris, pH 8.0; 1 mM EDTA) containing 1 mg/mL lysozyme (Sigma, Darmstadt, Germany) and total RNA was isolated from lysed cells using TRI reagent (Sigma) according to manufacturer’s protocol. Removal of DNA was achieved by treatment of samples with TURBO DNA-free kit (Thermo Fisher Scientific, Waltham, MA, USA). RNA quality and quantity were determined by agarose gel electrophoresis and using the Nanodrop 2000 machine (Thermo). Furthermore, the RNA quality was assessed at the sequencing facility of the Institute of Advanced Biotechnology (AIB) on an Agilent 2100 Bioanalyzer device. All samples displayed RNA integrity numbers higher than 9.

### 4.3. RNA-Seq and Data Analysis

Prior to sequencing, ribosomal RNA was depleted with Ribo-Zero rRNA removal kit for bacteria (Illumina, San Diego, CA, USA). Libraries were prepared with NEBNext Ultra II Directional RNA Library Prep kit (Illumina) and sequenced on a NovaSeq 6000 platform (Illumina) using paired-end 50 base-pair read protocol at AIB. RNA-seq data are deposited at the European Nucleotide Archive under project accession number PRJEB40950. Quality control of the obtained reads was done using FastQC (https://www.bioinformatics.babraham.ac.uk/projects/fastqc/). Quality trimming and adaptor removal were performed using trimmomatic [64]. Next, reads were mapped to *B. pertussis* Tohama I transcriptome and quantified using the Salmon algorithm [65]. Unwanted variation caused by batch or library preparation effects was removed using the RUVs method from RUVSeq [66], and differential gene expression analysis was performed using DESeq2 [67]. Genes with a |log_2_-fold change| ≥ 1 and with adjusted *p*-value ≤ 0.05 were considered as significantly differentially expressed. PCA plot for RNA-seq samples was prepared using the “plotPCA” function from the DESeq2 package. Heatmap showing the hierarchical clustering of the RNA-seq samples according to Euclidian sample-to-sample distances was visualized using pheatmap package in R. Enrichment analysis of gene ontology (GO) terms for significantly up- and downregulated genes was done as described before [19]. Briefly, each GO-term was tested for enrichment using Fisher’s exact test and *p*-values were corrected for multiple testing using the method of Benjamini and Hochberg. Enriched GO terms were summarized and visualized using Revigo [68].

### 4.4. Quantitative PCR (RT–qPCR)

For the RT–qPCR, duplicates of 0.5 μg of total isolated RNA treated with TURBO DNA-free kit (Thermo) were reverse transcribed into cDNA using M-MLV reverse transcriptase (Promega Madison, WI, USA) according to the manufacturer’s protocol. RT–qPCR was performed on Bio-Rad CFX384 Touch™ instrument using SYBR^®^ Green JumpStart™ Taq ReadyMix™ (Sigma), 5 pmoles of each primer and 40 ng of reverse-transcribed RNA in a 25 μL qPCR reaction with an initial step at 95 °C for 12 min, followed by 40 cycles of 95 °C for 15 s, 60 °C for 25 s, 72 °C for 30 s. Three qPCR reactions were done for each sample of reverse-transcribed RNA. The *rpoB* gene was used as a reference gene; all primers used for the RT–qPCR reactions were published before [16]. The relative gene expression was calculated using amplification efficiency values [69].

### 4.5. Protein Isolation and Sample Preparation for Proteomics

Filtered supernatants of *B. pertussis* cultures were precipitated with 10% (*w/v*) trichloracetic acid (Sigma) overnight at 4 °C. Precipitated proteins were collected by centrifugation (14,000× *g*, 4 °C, 20 min), washed with 80% acetone and finally dissolved either in sample buffer for immunoblot analysis or in TEAB digestion buffer (100 mM Triethylammonium bicarbonate, pH 8.5, 2% sodium deoxycholate) for LC–MS/MS. Protein concentrations were determined using a BCA protein assay kit (Thermo), and 20 µg of protein per sample were used for protein analysis. Cysteines were reduced with 5 mM Tris(2-carboxyethyl)phosphine (60 °C for 60 min) and blocked with 10 mM methyl methanethiosulfonate (10 min, room temperature). Samples were digested with trypsin (trypsin to protein ratio 1:20) at 37 °C overnight. Digestion of samples was stopped by the addition of trifluoroacetic acid (Sigma) to a final concentration of 1% (*v/v*). Sodium deoxycholate was removed by extraction with ethyl acetate, and peptides were desalted on the C18 column (Michrom Bio, Auburn, CA, USA).

### 4.6. LC–MS/MS and Data Analysis

Label-free LC-MS/MS analysis was performed as described elsewhere [19]. Raw data were imported into MaxQuant software (version 1.5.3.8) [70] for label-free quantification of proteins. The false discovery rate (FDR) was set to 1% for peptides and a minimum specific length of seven amino acids. The Andromeda search engine [71] was used for the MS/MS spectra search against the Uniprot *Bordetella pertussis* database (downloaded in November 2016). Protein abundance was calculated from obtained label-free protein intensities using the MaxLFQ algorithm [72]. For downstream analyzes, only proteins with more than four MS/MS spectral counts and which were detected in at least two of the three biological replicates were considered. In addition, the protein data set was reduced by potential and reverse contaminants and peptides identified only by the site. Statistics and data interpretation were performed using Perseus 1.6.2.3 software [73]. Prior to data analysis, we used the imputation method using sampling from a normal distribution with parameters robustly estimated from analyzed data. Each abundance ratio was tested for significance with Student’s t-test, non-paired. The *p*-values were further adjusted for multiple testing correction to control the false discovery rate at a cut-off of 0.1 using the permutation test (number of randomization 250). Proteins with corrected *p*-value (*q*-value) < 0.1 were considered as significantly modulated. The proteomics data were deposited to the ProteomeXchange Consortium via the PRIDE [74] partner repository with the dataset identifier PXD022760.

### 4.7. Protein Detection by Immunoblotting

Samples equivalent to 1.0 OD_600_ unit were separated on 12.5% SDS-polyacrylamide gels and transferred onto a nitrocellulose membrane using Trans-Blot^®^ Turbo™ system (Bio-Rad, Hercules, CA, USA). Gels were stained after the transfer to evaluate the transfer efficiency and also served as loading controls. Membranes were blocked with 5% skim milk and probed with in-house produced mouse polyclonal antibodies raised against Bsp22, BopN and BteA at a 1:10,000 dilution followed by incubation with anti-mouse IgG antibodies conjugated with horseradish peroxidase (Cell Signaling Technology, Inc., Danvers, MA, USA) at a 1:10,000 dilution. The antibody–antigen complexes were visualized using SuperSignal West Femto chemiluminescent substrate (Thermo) according to the standard protocol on G:Box Chemi XRQ device (Syngene, Cambridge, UK).

### 4.8. Construction of B. pertussis BP2259 Deletion Mutant

The deletion of the *BP2259* gene in *B. pertussis* Tohama I was performed using the pSS4245 allelic exchange system [75]. First, two DNA fragments corresponding to the upstream and the downstream regions flanking the *BP2259* gene were created using PCR. The upstream fragment containing the *Eco*RI and *Nhe*I sites (in italics) and start codon (underlined) of the gene was amplified using primers AATA*GAATTC*CTGATTCAGGCGCTGGGACACG and AAA*GCTAGC*CATGGCGTCACGCCTCCAGG while the downstream fragment, containing the *Nhe*I and *Eco*RI sites (in italics) and stop codon (underlined) of the gene was amplified using primers AAT*GCTAGC*AGCGCGCTCTACCAGGGATGAT and AATA*GAATTC*ATGGCGTCCAGCGAGAAGCG. Both PCR products were cleaved with *Nhe*I and ligated. The ligation mixture was used to amplify the ligated product, which was then cleaved with *Eco*RI and ligated into a pSS4245 vector cleaved with the same enzyme. Next, the resulting plasmid was transformed into *E. coli* SM10 strain and transferred into *B. pertussis* Tohama I by conjugation. After two recombination events, the strain carrying the markerless in-frame deletion of *BP2259* was obtained. The deletion was confirmed by DNA sequencing of the PCR-amplified chromosomal region.

## Figures and Tables

**Table 1:** List of genes significantly modulated in liquid cultures upon previous exposure to blood.

Gene ID	Name	log_2_FC ^1^	adj. *p*-Value	Function
BP0500	*bteA*	1.20	4.02 × 10^−12^	Type III secretion system protein BteA
BP1005		1.16	0.000134	Hypothetical protein
BP1226		1.21	0.006475	Hypothetical protein
BP1610		1.54	2.42 × 10^−5^	Pseudogene
BP1652		1.41	8.66 × 10^−38^	Pseudogene
BP2228	*alr*	1.02	2.57 × 10^−10^	Alanine racemase
BP2252	*bopB*	1.09	7.09 × 10^−6^	Type III secretion system protein BopB
BP2253	*bopD*	1.09	4.04 × 10^−6^	Type III secretion system protein BopD
BP2254	*bcrH1*	1.12	9.68 × 10^−6^	Type III secretion system chaperone BcrH1
BP2255	*btc22*	1.11	0.000178	Bsp22 chaperone Btc22
BP2256	*bsp22*	1.15	7.88 × 10^−6^	Type III secretion system tip protein Bsp22
BP2374		1.94	4.21 × 10^−13^	Hypothetical protein
BP2749	*putA*	1.89	0.0204	Proline dehydrogenase
BP3007		−1.01	4.99 × 10^−14^	Hypothetical protein
BP3011		−1.73	1.54 × 10^−22^	Hypothetical protein
BP3302		2.29	2.45 × 10^−13^	Hypothetical protein
BP3441		1.25	9.11 × 10^−8^	Hypothetical protein
Bpnc_071		1.11	2.70 × 10^−10^	Candidate_Transcript_071

^1^ Log_2_FC values of B0/A0 cell comparison are shown for RNA-seq analysis.

**Table 2:** List of proteins significantly differentially secreted upon previous exposure to blood.

Protein ID	FC ^1^	q-Value	Fasta Header
BP0200	2.14	0.022	Putative exported protein
BP0500	32.04	0	T3SS effector protein (BteA)
BP0760	3.68	0.039	Bifunctional hemolysin/adenylate cyclase
BP1722	−2.69	0.037	DNA_pol_B_exo2 domain-containing protein
BP1802	−3.94	0.097	Co-chaperone protein HscB homolog
BP2059	−3.33	0.077	Putative thiolase
BP2078	−1.08	0.034	Pyridoxine 5-phosphate synthase
BP2172	−2.73	0.097	Cbb3-type cytochrome c oxidase (BP2172)
BP2233	18.93	0.019	Uncharacterized protein (BtrA)
BP2252	57.30	0	T3SS outer protein B (BopB)
BP2253	33.80	0	T3SS outer protein D (BopD)
BP2255	1.20	0.065	Uncharacterized protein (BP2255)
BP2256	32.93	0	T3SS-secreted protein (Bsp22)
BP2257	38.72	0.027	T3SS outer protein N (BopN)
BP2259	27.11	0.031	Uncharacterized protein (Bp2259)
BP2440	−1.23	0.089	Acyl carrier protein
BP2514	1.78	0.097	Formyltetrahydrofolate deformylase (PurU)
BP2667	−2.64	0.036	Adhesin (FhaS)
BP3284	−4.07	0.040	ATP synthase subunit b
BP3434	1.47	0.024	Putative exported protein

^1^ FC values of B0/A0 culture supernatants comparison are shown for LC–MS/MS analysis.

## Data Availability

Not applicable.

## Supplementary Materials

Supplementary Materials can be found at https://www.mdpi.com/1422-0067/22/2/736/s1.

## Abbreviations

CHA	Charcoal agar
CHAB	Charcoal agar supplemented with blood
FDR	False discovery rate
TEAB	Triethylammonium bicarbonate
EDTA	Ethylenediaminetetraacetic acid
DE	Differential expression
SS	Stainer–Scholte
RT–qPCR	Quantitative reverse transcription PCR
PCA	Principal component analysis
GO	Gene ontology
LFQ	Label-free quantification
T3SS	Type 3 secretion system
FC	Fold change
