# Supplementary material for: Omics Analysis of Blood-Responsive Regulon in Bordetella pertussis Identifies a Novel Essential T3SS Substrate
Source: Int J Mol Sci. 2021 Jan 13;22(2):736. doi: 10.3390/ijms22020736 (PMC7828420; doi:10.3390/ijms22020736)
Supplement: Supplementary file 1 [file ijms-22-00736-s001.zip › Figure S1.pdf]

Drzmisek *et al*. Omics analysis of blood-responsive regulon in *Bordetella pertussis* identifies a novel essential T3SS substrate

Figure S1

```
T-COFFEE, Version_11.00 (Version_11.00)
Cedric Notredame
SCORE=994
*
 BAD AVG GOOD
*
Version_A   :  98
Version_B   :  99
Version_C   :  99
Version_D   :  99
cons        :  99

Version_A     MPSGLSCRHGTEEPKASHSAYSMFPLRRTRYTQGFETTAHRMNFQIPPALPALELDVFARAASQGETLY
Version_B     MEPKSRKRHTVHIP------YSMFPLRRTRYTQGFETTAHRMNFQIPPALPALELDVFARAASQGETLY
Version_C     MPSGLSCRHGTEEPKASHSAYSMFPLRRTRYTQGFETTAHRMNFQIPPALPALELDVFARAASQGETLY
Version_D     MPWGLSCRHGTEEPKASHSAYSMFPLRRTRYTQGFETTAHRMNFQIPPALPALELDVFARAASQGETLY

cons          *      ** .. *      **********************************************


Version_A     VTKAGEQFQVIASGTTPSGRNVSWVATDEDTLVMFSSALALAYGTGIARAVKELDLHAVPTTSLSARV
Version_B     VTKAGEQFQVIASGTTPSGRNVSWVATDEDTLVMFSSALALAYGTGIARAVKELDLHAAPTTSLSARV
Version_C     VTKAGEQFQVIASGTTPSGRNVSWVATDEDTLVMFSSALALAYGTGIARAVKELDLHAAPTTSLSARV
Version_D     VTKAGEQFQVIASGTTPSGRNVSWVATDEDTLVMFSSALALAYGTGIARAVKELDLHAAPTTSLSARV

cons          **********************************************************.*******


Version_A     VTRAVDMAETSRHALQGVDFLTFLSWSARADAAGFRQVCHDTGVSPDQISGTLRATIDESMQQRFASAA
Version_B     VTRAVDMAETSRHALQGVDFLTFLSWSARADTAGFRQVCHDTGVSPDQISGTLRATIDESMQQRFASAA
Version_C     VTRAVDMAETSRHALQGVDFLTFLSWSARADTAGFRQVCHDTGVSPDQISGTLRATIDESMQQRFASAA
Version_D     VTRAVDMAETSRHALQGVDFLTFLSWSARADTAGFRQVCHDTGVSPDQISGTLRATIDESMQQRFASAA

cons          *******************************:**********************************


Version_A     QSGKAPVSAHTAQEWLREVLAHHLV
Version_B     QSGKAPVSAHTAQEWLREVLAHHLM
Version_C     QSGKAPVSAHTAQEWLREVLAHHLM
Version_D     QSGKAPVSAHTAQEWLREVLAHHLM

cons          ************************:
```

BtrA versions:

Version A: all *B. pertussis* strains + *B. bronchiseptica* strains FDAARGOS_693, A310, A345, F709, FDAARGOS_634, I943.

Version B: *B. bronchiseptica* strains RB50, D973, FDAARGOS_176, S798.

Version C: *B. bronchiseptica* strains D448, E001, E016, KVNON-570, NCTC10543, NCTC8344.

Version D: *B. bronchiseptica* strains ATCC 10580, D987, 253, E010.

FASTA sequences:

```
>Version_A
MPSGLSCRHGTEEPKASHSAYSMFPLRRTRYTQGFETTAHRMNFQIPPALPALELDVFARAASQGETLYV
```

TKAGEQFQVIASGTTPSGRNVSWVATDEDTLVMFSSALALAYGTGIARAVAKELDLHAVPTTSLSARVVT
RAVDMAETSRHALQGVDFLTFLSWSARADAAGFRQVCHDTGVSPDQISGTLRATIDESMQQRFASAAQSG
KAPVSAHTAQEWLREVLAHHLV

>Version_B
MEPKSRKRHTVHIPYSMFPLRRTRYTQGFETTAHRMNFQIPPALPALELDVFARAASQGETLYVTKAGEQ
FQVIASGTTPSGRNVSWVATDEDTLVMFSSALALAYGTGIARAVAKELDLHAAPTTSLSARVVTRAVDMA
ETSRHALQGVDFLTFLSWSARADTAGFRQVCHDTGVSPDQISGTLRATIDESMQQRFASAAQSGKAPVSA
HTAQEWLREVLAHHLM

>Version_C
MPSGLSCRHGTEEPKASHSAYSMFPLRRTRYTQGFETTAHRMNFQIPPALPALELDVFARAASQGETLYV
TKAGEQFQVIASGTTPSGRNVSWVATDEDTLVMFSSALALAYGTGIARAVAKELDLHAAPTTSLSARVVT
RAVDMAETSRHALQGVDFLTFLSWSARADTAGFRQVCHDTGVSPDQISGTLRATIDESMQQRFASAAQSG
KAPVSAHTAQEWLREVLAHHLM

>Version_D
MPWGLSCRHGTEEPKASHSAYSMFPLRRTRYTQGFETTAHRMNFQIPPALPALELDVFARAASQGETLYV
TKAGEQFQVIASGTTPSGRNVSWVATDEDTLVMFSSALALAYGTGIARAVAKELDLHAAPTTSLSARVVT
RAVDMAETSRHALQGVDFLTFLSWSARADTAGFRQVCHDTGVSPDQISGTLRATIDESMQQRFASAAQSG
KAPVSAHTAQEWLREVLAHHLM
